# High-Throughput Screening Platforms in the Discovery of Novel Drugs for Neurodegenerative Diseases

**DOI:** 10.3390/bioengineering8020030

**Published:** 2021-02-23

**Authors:** Hasan Aldewachi, Radhwan N. Al-Zidan, Matthew T. Conner, Mootaz M. Salman

**Affiliations:** 1 Biomolecular Sciences Research Centre, Sheffield Hallam University, Sheffield S1 1WB, UK; hsaldewachi@uomosul.edu.iq; 2 College of Pharmacy, Nineveh University, Mosul 41002, Iraq; 3 College of Pharmacy, University of Mosul, Mosul 41002, Iraq; radhwan.alzidan@uomosul.edu.iq; 4 School of Applied Sciences, Edinburgh Napier University, Edinburgh EH11 4BN, UK; 5 School of Sciences, Research Institute in Healthcare Science, University of Wolverhampton, Wolverhampton WV1 1LY, UK; m.conner@wlv.ac.uk; 6 Oxford Parkinson’s Disease Centre, Department of Physiology, Anatomy and Genetics, University of Oxford, South Parks Road, Oxford OX1 3QX, UK

**Keywords:** high-throughput screening, HTS, neurodegenerative diseases, drug discovery, dementia, brain diseases, CNS disorders, tauopathies, bioassays, dementia

## Abstract

Neurodegenerative diseases (NDDs) are incurable and debilitating conditions that result in progressive degeneration and/or death of nerve cells in the central nervous system (CNS). Identification of viable therapeutic targets and new treatments for CNS disorders and in particular, for NDDs is a major challenge in the field of drug discovery. These difficulties can be attributed to the diversity of cells involved, extreme complexity of the neural circuits, the limited capacity for tissue regeneration, and our incomplete understanding of the underlying pathological processes. Drug discovery is a complex and multidisciplinary process. The screening attrition rate in current drug discovery protocols mean that only one viable drug may arise from millions of screened compounds resulting in the need to improve discovery technologies and protocols to address the multiple causes of attrition. This has identified the need to screen larger libraries where the use of efficient high-throughput screening (HTS) becomes key in the discovery process. HTS can investigate hundreds of thousands of compounds per day. However, if fewer compounds could be screened without compromising the probability of success, the cost and time would be largely reduced. To that end, recent advances in computer-aided design, in silico libraries, and molecular docking software combined with the upscaling of cell-based platforms have evolved to improve screening efficiency with higher predictability and clinical applicability. We review, here, the increasing role of HTS in contemporary drug discovery processes, in particular for NDDs, and evaluate the criteria underlying its successful application. We also discuss the requirement of HTS for novel NDD therapies and examine the major current challenges in validating new drug targets and developing new treatments for NDDs.

## 1. Introduction

High-throughput screening (HTS) has increasingly been used for novel drug discovery in the field of pharmaceutics replacing the traditional “trial and error” approach to identify therapeutic targets and validate biological effects [1,2,3]. HTS involves assaying and screening a large number of biological effectors and modulators against designated and exclusive targets. Thus, HTS is generally favored when little is known of the target, which precludes structure-based drug design, but it can also be used in parallel with other strategies such as computational techniques and fragment-based drug design [4,5]. HTS comprises several steps including target recognition, compound management, reagent preparation, assay development, as well as the screening itself. Using HTS in conjunction with multi-well cell-based platforms allows for the identification of small molecule modulators of related biochemical and signal transduction pathways.

**Figure 1:**
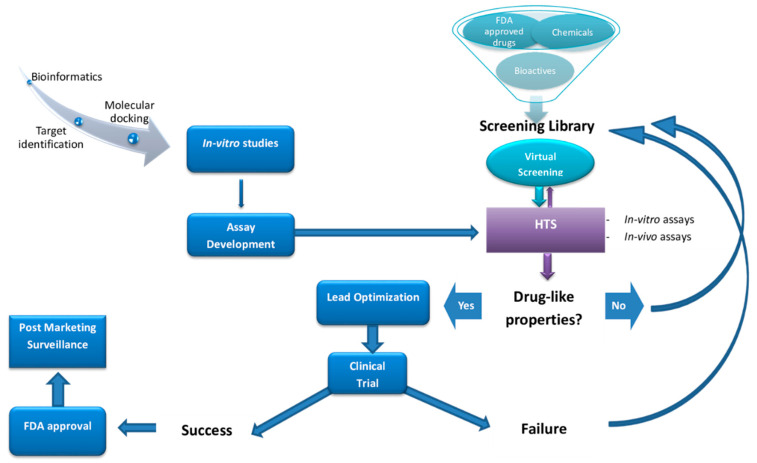
Steps involved in the process of drug discovery.

Current programs for central nervous system (CNS) drug development and discovery can be subdivided into four main areas: (1) Receptor and target engagement, (2) drug “hit” identification, (3) lead identification, and (4) drug lead optimization. Active compounds resulting from HTS screens—the so-called “hits”— are the prototypes from which drug “leads” are ultimately formed through additional combinatorial and medicinal chemistry. Following the screening of several hundred thousand small-molecules, a few hundred “hits” may be identified, leading potentially to viable drug compounds (Figure 1). Potential hits from the HTS must then be configured for delivery, metabolism, and pharmacokinetics to suggest initial lead compounds. However, these lead compounds generally require considerable medicinal chemistry optimization, for example, to block polar functional groups that may reduce high receptor affinity, in an effort to generate a medicine that has optimal drug metabolism and pharmacokinetic (DMPK) properties.

Neurodegenerative diseases (NDDs) are incurable and debilitating conditions that result in progressive degeneration and/or death of nerve cells in the CNS [6,7,8]. Developing drugs for CNS disorders; in particular NDDs, has become a risky business, where most candidates fail after years of costly clinical and non-clinical related activities. Thus, one of the critical first steps in the advancement of treatments of NDDs is the development of accurate assays for investigating neurodegeneration [9]. While the word “neurodegeneration” can be applied to a wide range of characteristics that cause the loss of neuronal activity, neuronal death is the most direct and prominent indicator of neurodegeneration. 

The creation of successful assays includes the ability to identify the associated events that trigger and cause cell death. To this end, tests have been established to recognize biochemical events that contribute to neuronal death such as metabolic fluctuation, energy metabolism [10], and fragmentation of DNA [11]. Cytoprotective assays using dyes or fluorescent markers have been a crucial method in the past to classify therapeutics causing neuronal death [11,12,13]. Many of these tests have the benefit of being easily suited to HTS systems and are frequently used by pharmaceutical companies to investigate the neurotoxicity of drugs and their possible side effects. HTS in primary neurons combines the advantages of HTS with the biological importance of being able to capture critical cell events or homeostatic conditions that are present in disease states. Despite being difficult to transfect and requiring complicated culture protocols, HTS with primary neurons is still feasible, with increased biological and clinical relevance being worth the extra effort and expense [14]. For example, Sharma et al. (2013) developed a HTS method for primary neurons which is applicable for large-scale testing, ranging from compound libraries to whole-genome RNA interference (RNAi) [15].

Novel diagnostic technologies for temporal analysis of the neuronal region and consistency of the cell membrane have been developed, providing assays to track neurodegeneration over time [16]. Neurons may become defective in certain NDDs long before they die [17]. The detection of specific disease-related impairment, especially prior to associated cell death, is therefore an important step forward in the discovery of therapeutics. In the case of Amyotrophic lateral sclerosis (ALS) and Alzheimer’s disease (AD), for example, neurons in the nervous system’s most susceptible cell populations have been shown to become overactive years before noticeable clinical signs and neuropathology have been identified [18,19,20]. There are a number of abnormal characteristics and symptoms associated with NDDs, therefore, a major challenge remains to have a reliable screening phenotype when detecting complex disease-related signatures that can be distinct and predictive of disease and cell death.

In this review, we discuss the current challenges in validating new drug targets and developing new treatments for NDDs. Moreover, we review the increasing role of HTS in the drug discovery process focusing on existing platforms that mimic healthy and diseased states of the CNS. In addition, we identify the main strengths and limitations in their applications towards identifying new therapeutic targets and therapeutics for NDDs.

Druggable targets are scanned either virtually utilizing virtual compound structure libraries and/or by cell-based or biochemical testing of available peptide or chemical libraries via high-throughput screening (HTS). Abbreviations: Food and Drug Administration (FDA).

## 2. High-Throughput Screening (HTS)

### 2.1. Formats and Major Considerations for HTS Platforms

HTS involves in vitro, cell- or whole organism- based assays [21]. The most common readouts for biochemical assays in HTS are optical, including absorbance, fluorescence, luminescence, and scintillation. The efficiency of data production and cost per screen are the main determinants in the choice of the most suitable readout for a particular screen. However, the fluorescence-based techniques are considered as one of the primary detection methods [22]. This can mainly be attributed to the high sensitivity and diverse range of available fluorophores enabling multiplexed readouts which allow miniaturization, assay design stability, ease of handling, and the ability to simultaneously track several events in real time [23]. However, it is important to note that short wavelength excitation (particularly those under 400 nm) should be avoided during the development of functional assays in order to reduce interference from test compounds [24,25,26]. This direct screening approach has been applied to the selection of thrombin inhibitors, HIV-protease inhibitors, DNA gyrase inhibitors, etc. [27,28,29]. Quantitative kinetics of compound binding can be used to gain a higher level of understanding of binding mechanisms, as it is possible to investigate the effect of structural variations in a systematic way. Association and dissociation rates can vary independently for a specific lead series, resulting in the rapid evolution of sub nanomolar-affinity leads [30].

Data from screens can be archived and reviewed using information management systems [31] or more laboriously, in Excel spreadsheets. The data is evaluated in order to classify hits: Data points that surpass a certain specified threshold to determine a positive result. Importantly, the threshold limits can be quite subjective, but a value of three standard deviations from the mean signal of wells treated with DMSO, for example, is a fair and typical cut-off, since it offers a manageable false-positive statistical hit rate (0.15%) [32]. Alternatively, the maximum number of hits that can be processed may be increased by “cherry picking”, normally several hundred compounds can be simply picked for further evaluation. Additionally, the median rather than the mean for a single compound can be used to assess hits if the screening is done in triplicate together with the use of appropriate statistical methods [32]. This protects against the undue influence of significant outlier results, which are common in these techniques. 

### 2.2. Main Types of HTS Assays

#### 2.2.1. Cell-Based Assays

Using cell-based assays, whole pathways can be investigated generating numerous potential points of interest, as opposed to the analysis of particular predetermined steps as in biochemical assays. Moreover, cell-based assays may provide data that cannot be obtained from a biochemical assay, such as the existence of the pharmacological activity of the screened compound at a particular receptor or the intracellular target [33,34]. Consequently, cell-based platforms are especially promising as important tools in the study of cell growth and differentiation, in examining the influence of small molecules and cell growth conditions on cell function and physiology, and also in understanding signaling pathways in mammalian cells. They have also proven to be particularly valuable in studying complex conditions such as CNS injury and NDDs, as many factors can contribute to a specific cellular response [35]. 

HTS is frequently accomplished using scaled down cell-based methods. Cell-based tests enable chemical libraries to be tested for molecules that exhibit a diversity of biological activities. In the pharmaceutical industry, cellular microarrays utilizing 96- or 384-well microtiter plates with 2D cell monolayer cultures are commonly used [35]. Cellular microarrays consist of a solid framework wherein minute volumes of diverse biomolecules and cells can be presented, permitting the multiplexed examination of living cells and, subsequently, the assessment of cellular reactions [33,36]. Miscellaneous molecules such as antibodies, polymers and small molecules can be arrayed using automated spotting technology or soft lithography [37]. Cellular microarrays are also used in small molecule screening [38,39]. The screening of small molecules in mammalian cell lines, such as CHO cells, could be considered as an example of utilization of such a system [40,41]. There is flexibility in choosing the readout when using a cell-based assay focused on a signaling pathway. For example, if an antibody is available, every stage in which a protein is modified (e.g., phosphorylated), translocated [42] or changed in its abundance [43,44,45] can be possible readouts [46]. Multiple NDDs have been studied both with target-based and cell-based screens, including AD [47], PD [48], bipolar disease, autism and schizophrenia [49]. A key feature of cell-based screening is that multiple targets are screened at once, the readout being the outcome of a cellular pathway or network [50].

#### 2.2.2. Biochemical Assays

Biochemical screening utilizes a purified target protein of interest and measures the binding of ligands or the inhibition of enzymatic activity in vitro [51]. These assays are generally conducted in a competition format, in which the compound under study displaces a known ligand or substrate. These assays are typically conducted in 384-well plates, which provide a good compromise between screening volumes (20–50 µL), throughput, and the cost of more sophisticated screening equipment. The readout is typically an optical method such as absorbance, fluorescence or luminescence [52]. Buratti et al. developed a method in which the activity of a specific RNA binding protein (RBP) (TDP-43) was measured, and due to the established activity of this protein, RBP was shown to be involved in the pathology of PD, AD, and other NDDs [53]. Additionally, Crowe et al. performed a novel study, screening almost 300,000 compounds to evaluate their effect on tau protein assembly. Formation of toxic tau oligomers in the brain is one of the main observed pathologic events of AD [54]. Using HTS assays based on complementary thioflavin T fluorescence and fluorescence polarization methods, the effects of inhibitors of tau oligomerization were determined. Specifically, that aminothienopyridazines (ATPZs) caused the inhibition of fibril assembly as well as fibrillization of tau. Additionally, the normal ability of tau to stabilize microtubules was not affected and ATPZs were shown to be promising drugs to treat AD [54,55]. Scaling down of bioanalytical activities, in order to decrease production expenses, as well as simplifying transport and saving space in the laboratory has led to a focus on laboratory-on-a-chip technology. Overall, scaling down improves the efficiency of required screening [56,57]. However, this could be complicated by extensive time implications, error-recovery rates, and complex experimental design often involving an error-prone robotic operation. 

**Figure 2:**
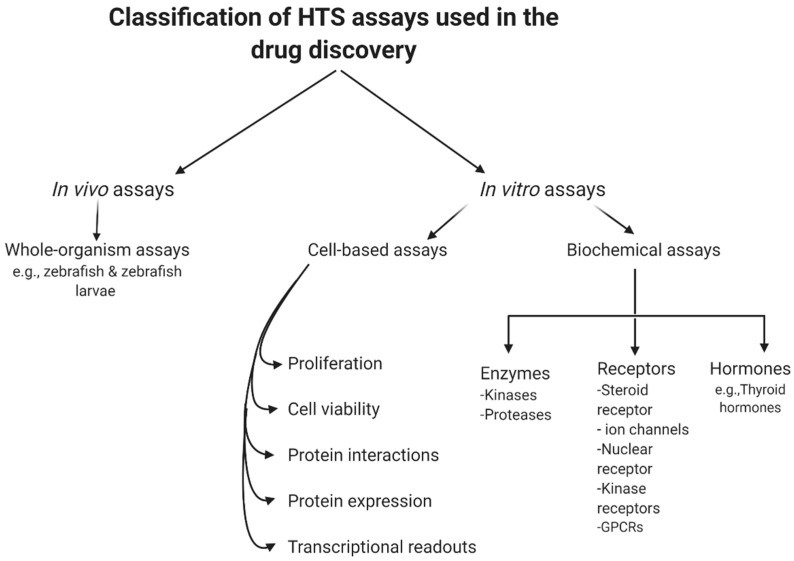
General classification of high-throughput screening (HTS) assays.

In summary, biochemical assays have the advantage that all hits found are against a known target by design. However, in those situations, the often costly and tedious determination of the molecular mechanisms of action would be needed, even though the target is known. Furthermore, due to the degree to which the predicted target was initially validated in the disease phase, the therapeutic potential of an in vitro hit can still be inconsistent. Even following the determination of such mechanistic details, it is difficult to predict the behavior of such compounds in a more complex cellular environment, due to variability in cellular permeability and metabolism, toxicity, selectivity, and the potential off-target activity of the compound [58]. However, cell-based assays have the benefit of detecting compounds that affect a phenotype in a complex cellular environment, but still suffer from a poor understanding of the target and mechanism of action. In addition, these experiments are usually more expensive and difficult to conform to miniature HTS assays [9]. Figure 2 summarizes the current classification of the main HTS assays.

### 2.3. Economics of HTS

**Figure 3:**
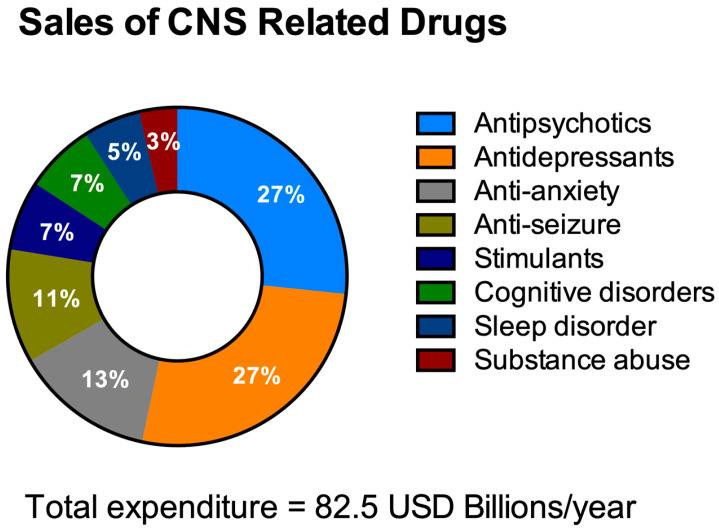
Total expenditure on central nervous system (CNS)-related drugs in 2010. Adapted from Gustavsson et al., 2011 [77].

HTS aims to decrease the costs of drug invention [59,60]. It is necessary to address the economics of HTS for NDDs drug discovery especially with the escalating yearly costs of mental and neurological pathologies (estimated to be around USD 1 trillion [61]) including drug sales figures (Figure 3). It is remarkable to note that 40% of these total costs were attributable to the lack of productivity of the affected population due to the presence of these diseases [62]. The financial burden of these pathologies is only likely to increase as they typically have long-term consequences combined with an increasingly aging population. 

It is crucial to extensively enhance our knowledge and understanding of CNS diseases in order to be able to develop effective therapies. Interestingly, despite the number of individuals in the US who experience CNS disorders being more than double than people who suffer from cardiovascular diseases (CVDs), the global market for CNS therapeutics constitutes less than a third of the global drug market for CVDs [63]. Therefore, the CNS drug market would have to increase by over 5-fold just to correspond to the global market for CVDs. 

The primary explanation for this under-development of the worldwide brain drug market is that the vast majority of CNS drugs do not cross the in vivo blood-brain barrier (BBB). The BBB is a unique and highly selective vascular interface that separates the peripheral blood circulation from the neural tissue in order to maintain an optimum homeostatic microenvironment for brain function and protection [64,65]. However, biology’s proverbial double-edged sword means that the protective nature of the BBB precludes almost all large-molecule neurotherapeutics and more than 98% of all small-molecules as viable drugs [66]. In one systematic medicinal research study, over 7000 drugs were evaluated in the comprehensive medicinal chemistry (CMC) database [67] and this suggested that the CNS was affected by just 5% of these medications. In another study, only one out of every eight medicines analyzed were active in the CNS and only 1% of the total number of drugs were clinically active in the CNS for diseases [68].

The procedure involved in developing a new drug is an elaborative effort which is often a costly and lengthy process. On average, the cost of developing a new medicine is around USD 1.3 billion (2018) [69]. However, the expenditure of the research and development (R&D) departments of the major pharmaceutical companies can be as high as USD 2.87 billion (2013) to discover and test a new drug [70]. Despite these huge investments in new treatments targeting NNDs and an expanding pipeline, there have been more failures and setbacks than overall treatment successes. The failure rate of clinical trials for new treatments targeting NDDs, for example AD, is exceptionally high and usually exceeds 99% [71]. For example, during the period 2010–2015, all the clinical trials of potential medicines for treating AD failed and were terminated after reaching phase three [72]. Recently, Biogen terminated both of the phase III, ENGAGE (NCT02477800), and the EMERGE (NCT02484547) clinical trials of Aducanumab [BIIB037], since it failed to demonstrate a superior activity compared to the placebo [73,74,75]. Consequently, Biogen lost more than 5 years and USD 2.5 billion on the failed experimental drug Aducanumab [BIIB037] [74]. It is clear that R&D expenditures over time have the most impact on the overall cost of drug development [76].

## 3. Drugs Discovery for NDDs

### 3.1. Challenges in the Discovery of CNS Drugs

CNS drugs face substantial developmental obstacles relative to non-CNS drugs, largely due to a limited understanding of the complex pathophysiology of many of the diseases they aim to treat, along with difficulties in identifying and assessing acceptable clinical endpoints. Approving a new drug for CNS diseases typically faces additional burdensome regulations. For instance, a study published by the Tufts Centre for the Study of Drug Development (Tufts CSDD) highlighted a real problem that hampers the discovery and subsequent development of CNS drugs. The study found that for the period 1995–2007, success rates for CNS drugs were less than half of non-CNS drug approval rates (6.2% vs. 13.3%, respectively). Additionally, between 2000 and 2017, the time for approval, after submission of a marketing application for CNS drugs, was 38% longer than for non-CNS drugs [78]. In 2017, Gribkoff and Kaczmarek analyzed the approval period and approval rates of clinical projects investigating 274 CNS and 1168 non-CNS drugs, in which 42 CNS and 345 non-CNS compounds finally got approved by the United States Food and Drug Administration (USFDA) [79]. Furthermore, a new Tufts CSDD report also revealed that the total time required for the development process was 20% higher for CNS drugs, and that the number of CNS drugs given the FDA priority review was considerably lower compared to the non-CNS drugs [80]. 

As a result, CNS drug research and development projects have been exposed to major layoffs and eliminations over the last decade. Although there has been recent revival of interest in CNS drug discovery, past shifts in the priorities of the pharmaceutical and biotech industries represent the well-documented reality of CNS-drug discovery projects. CNS drugs in general have higher failure rates than non-CNS drugs, both preclinically and clinically, and in certain cases, such as for the main NDD disorders, the clinical failure rate for disease-modifying medications has been 100% [37]. Compared to non-CNS drugs, the development periods for CNS drugs are slightly longer for those drugs which are approved, and post-development regulatory approval is also longer [37,60]. 

Although the last few decades have witnessed major developments in our understanding of basic neuroscience, such as neuropharmacology, most CNS pharmaceutical treatments are distinguished not by the treatment of the cause but rather of symptoms. For example, most pain drugs (used in the CNS disease treatment) minimize the discomfort, but do not permanently influence the cause of pain. This is often acceptable for acute pain, especially when the cause is self-limiting, but when the drug is withdrawn chronic and neuropathic pain often returns. The treatment of symptoms, even if followed by severe side effects, can be very effective in psychiatric conditions, but when the drug is stopped, the symptoms usually return without a decrease in severity [81]. The general degeneration (death) of neurons in AD or the more localized deaths of particular central cell populations in PD and ALS, contribute to increased impairment and eventual death in people. Currently, all of the approved treatments for these chronic NDDs are palliative and symptomatic therapies.

### 3.2. The Need for HTS in the Discovery of Drugs for NDDs

NDD, also known as “protein-misfolding disorders”, are a heterogeneous group of diseases characterized by extensive neuronal loss, cellular toxicity, and cell proteostatic impairment. Extensive neuropathological, biochemical, and molecular genetic studies indicate that the accumulation of proteins with altered physical and chemical characteristics is a fundamental phenomenon in many forms of NDDs in the human brain, as well as in peripheral organs [82,83].

**Figure 4:**
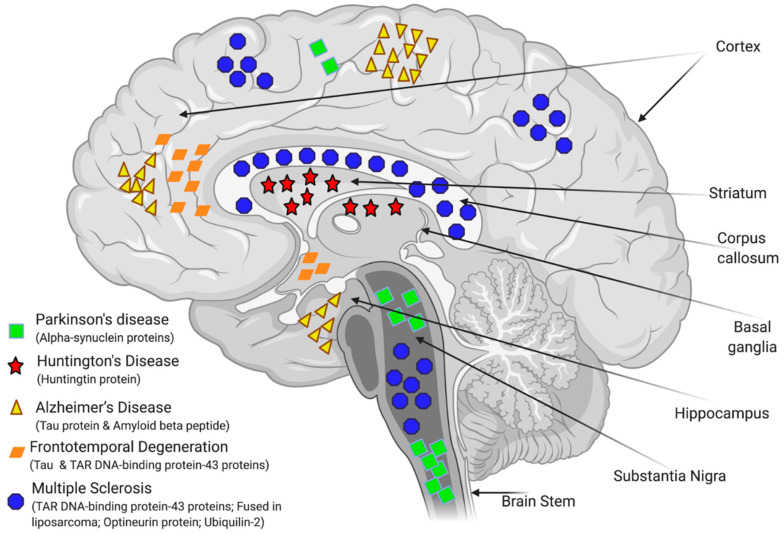
Schematic summary of the regions of the brain affected by major neurodegenerative diseases (NDDs) and the misfolded proteins that are involved in their pathology. Adapted from [90,91].

The precise aetiology of the majority of NDDs is highly complicated and not fully understood. Studies performed in the last few decades have shown that abnormal protein folding and deposition is a common characteristic within the different types of NDDs (see Figure 4). For instance, the tau protein, FUS (fused in liposarcoma)/FET protein (FUS/FET), TAR DNA-binding protein 43 (TDP-43), and alpha-synuclein protein (α-syn) can accumulate intracellularly in the nervous system. Whereas, amyloid beta protein (Aβ) or prion protein (PrP) are examples of proteins that can accumulate extracellularly and also lead to NDDs [84,85]. AD is characterized by the presence of hyperphosphorylated and misfolded intraneuronal aggregates of tau protein, and by the extracellular deposition of amyloid plaques. Lewy body (LB)-associated diseases, which include PD and Lewy body dementia (LBD), display intraneuronal cytoplasmic inclusions. Whereas the sporadic, adult-onset degenerative motion disorder of unknown aetiology, known as multiple system atrophy (MSA) is characterized by a pathological aggregation of toxic forms of α-syn within oligodendrocytes and neurons. Deposition of neuronal tau is an important feature of AD, frontotemporal lobar degeneration (FTLD), primary age-related tauopathy (PART), neurofibrillary tangle (NFT)-dementia, and pick disease (PiD). Argyrophilic grain disease (AGD), progressive supra-nuclear palsy (PSP), and cortico-basal degeneration (CBD) all show both neuronal and glial tau aggregates, whereas globular glial tauopathies (GGT) are characterized by glial tau disorders [86,87]. Moreover, recent advances have demonstrated that glial cells (including astrocytes, oligodendrocytes, and microglia) are involved in mediating the pathophysiology of various CNS disorders including NDDs by activating neuroinflammation and disrupting the BBB function [88], thereby affecting brain water homeostasis [44] and impairing brain energy metabolism [89], all of which ultimately contribute to neuronal death and neurodegeneration (Figure 4).

The discovery and development of any new drug relies heavily on a detailed understanding of the underlying mechanisms of disease and a successful progression from the identification of candidates to the design of clinical trials [92]. However, our current knowledge and understanding of the precise aetiology of the majority of the NDDs is still incomplete. Even animal models recapitulate only limited aspects of each disease. The extent to which they can model human diseases involving complex and poorly defined factors is still limited and unclear due to differences in anatomy and physiology and hence, pathophysiological responses involved in the disease process [93,94,95]. This might partially explain the high failure of a large number of lead compounds during the in vivo part of clinical trials [96,97]. Lead compounds with promising safety and efficacy profiles can still fail during in vivo stages due to various physiochemical properties, for example, failing to cross the BBB due to poor lipophilicity, charge, hydrodynamic radius, and/or molecular weight. Therefore, it is not surprising that an extremely small number of drug candidates, which proceed from preclinical and animal studies to phase I clinical trials, have eventually made it to the bedside [98].

Only a small class of drugs actually cross the BBB which includes small molecules with high lipid solubility and a low molecular weight (Mw) of <400–500 Daltons (Da). However, There are a few brain diseases that reliably react to this category of small molecules, including depression, affective disorders, chronic pain, and epilepsy [67,99]. The classic experiment of intravenous injection of [^14^C]-histamine into mice has demonstrated the rate-limiting function of the BBB. Histamine is a small molecule of just 111 Da, but does not cross the BBB due to the presence of many hydrogen-bond-forming functional groups. In addition, BBB penetration is known to be inversely related to the number of hydrogen bonds (typically <8 hydrogen bonds to be able to cross BBB) [100]. Nevertheless, the vast majority of CNS drugs that evolved from HTS are either water soluble with a high degree of hydrogen bonding or have a Mw of more than 500 Da. Applying HTS to the discovery of CNS drugs has led to an increase in the molecular weight of drugs and a decrease in the lipid solubility of drugs. Without HTS, large molecular weight medicines would not have been developed for the CNS due to the BBB selectivity. In fact, potential large-molecular weight drugs that are found to be effective in the brain may still be neglected in favor of a quest for peptidomimetic small molecules [66]. Except for some endogenous ligands which are already small molecules, no small molecule peptidomimetics have been discovered to date that are able to transport through the BBB [66].

It is widely accepted that toxic misfolded proteins potentially underlie many NDDs. However, individual targets that regulate these proteins and their detrimental downstream effects are still not fully understood nor established. Phenotypic screening is an objective approach for identifying new targets and therapeutic molecules spanning a wide range of models from primitive organisms such as *S. cerevisiae* to more pathophysiologically relevant patient-derived cellular models.

The HTS of small molecules enables a rapid analysis of the effects of thousands or even millions of small molecules. Hence, it could be highly rewarding to exploit HTS in the field of drug discovery for NDDs. For example, AD is high on the list of leading causes of death in the United States and worldwide and therefore, there is a significant global market for disease-modifying medications for AD [101]. The number of people afflicted by AD is expected to increase dramatically as the “baby boom” generation ages and medical advances allow more people to live longer. For instance, in 2015, it was estimated that there is a new case of AD every 65 s in the US. However, by 2050, a new case of AD is predicted to occur every 33 s, or approximately 1,000,000 new cases every year [101,102]. Despite the presence of a number of FDA approved drugs to treat AD, such as galantamine, memantine, donepezil, tacrine, and rivastigmine, these drugs provide only symptomatic control. Lowering the AD prevalence rate and decelerating its progression will require new drugs that address the underlying pathophysiology of AD at a molecular level [93]. Therefore, drug discovery in the arena of NDDs will hugely benefit from efficient, rapid, and cost-effective approaches such as HTS to accelerate the discovery of potential new drugs for the increasing cases of NDD globally.

### 3.3. Modelling of NDDs for HTS

The ethical and financial concerns, time, and labor-intensive complexity of animal trials together with the obstacles associated with amending these models to the requirements of HTS have curtailed the wide use of such models for preliminary drug screening assays. Furthermore, the reported failure of many clinical trials addressing NDDs has raised doubts on the relevance of animal disease models to humans and implied the need for superior research tools [71,103,104,105,106]. Nonetheless, a number of in vivo models, such as Zebrafish, *Drosophila melanogaster*, and *Caenorhabditis elegans*, have been successfully used in modelling NDDs.

Zebrafish (*Danio rerio*) is a commonly used in vivo model for different NDDs [107,108]. Owing to their rapid development, small size, susceptibility to genetic manipulation, large numbers of offspring, and transparency during development, zebrafish are a useful model for advanced imaging [109,110,111]. Therefore, it has become an increasingly important in vivo model (over the traditional use of mammals) for HTS and effective in the determination of new compound safety and efficacy [112,113]. Zebrafish, however, have some inherent limitations when it comes to NDD modelling. For example, their CNS undergoes continuous growth and life-long development of new neurons, and displays remarkable potential for axon and neuron regeneration following focal lesion. This potential for continued development and repair may adversely affect their potential use as a model for certain aspects of neurodegeneration in humans [114,115,116].

Similarly, *C. elegans* offer an effective in vivo model for HTS [117,118,119]. The major advantages of *C. elegans* are their rapid growth rate and their cost-effectiveness as a model for the detection of compounds that have a protective effect(s) against the harmful impacts of the accumulated misfolded proteins in the neurons of patients suffering from NDDs such as ALS [120], Huntington’s disease (HD) [121], PD [122], and AD [123,124]. Another example is *Drosophila melanogaster* [125,126], which has similar advantages offered by zebrafish and *C. elegans* such as the rapid growth rate, amenability for genetic modifications, and successful application as a model of NDDs in HTS [127,128,129]. However, all of these models suffer from a common major drawback, the limited ability to effectively mimic the complex pathophysiological environment of NDDs in humans. For instance, all the above-mentioned models have a short lifespan which may lessen their relevancy in model lineage-related diseases in humans [130]. Moreover, *C. elegans* have a simplistic body plan, lack several defined tissues/organs such as blood, brain, and internal organs, and are evolutionarily distant from humans [131]. Moreover, *Drosophila melanogaster* do not have an adrenergic system [132]. Therefore, developing novel, and more physiologically relevant in vitro models, can bridge the gap between existing pre-clinical and human studies. 

Human embryonic stem cells (ESCs) and subsequently human induced pluripotent stem cells (iPSCs) have emerged as powerful tools due their ability to be reprogrammed into several distinctive tissue-specific cell types making them an ideal model for connecting phenotype to genotype [133,134,135]. An increasing line of evidence suggests the successful implementation of iPSC-based models in HTS assays for discovering drug candidates for NDDs [136]. The introduction of CRISPR-Cas9 technology has revolutionized genome-editing through providing a more effective, cheaper, and faster technique than the earlier methods [137]. It has facilitated the generation of cell-based specific assays for various NDDs using genetically modified (knock-in and knockout) iPSCs [132,135,136]. Moreover, CRISPR-Cas9 has opened the door for developing humanized animal models of NDDs. Currently, humanized animal models for PD [138], AD [139], ALS [140], and HD [141] have been successfully generated. These models have become favored in contemporary studies to test promising drug candidates which have successfully passed the in vitro validation [142].

Advanced 3D culture models using hydrogels, extracellular matrix (ECM) scaffolds, spheroids, organoids or organ-on-a-chip models have been developed to emulate the physiological environment and functionality of human organs which can be lost in traditional 2D models [143,144,145]. 3D culture models of the brain, BBB, or brain spinal cord barrier (BSCB) have been recently validated for various NDDs [146]. These models promote multicellular tissue-like formation with distinctive cell–cell and cell–matrix interactions required for near physiological functionality [147]. Three-dimensional cell culture models allow for a more precise prediction of the effects of potential drugs by closely recapitulating essential facets of the brain environment, mimicking neuronal and glial cell interactions, and integrating the effect of physiological blood flow, unidirectionality and access to oxygen and nutrients [148]. One of the major exciting steps in HTS is the development and implementation of the organ-on-a-chip model. The organ-on-a-chip model is a miniaturized microfluidic perfusion tool that enables the in vitro culture of primary cells or tissues for an extended duration in a format which is applicable for high throughput research. These models not only preserve the cell-cell interactions and capture key structural and functional aspects of organs, but they also enable the use of very limited quantities of investigational drugs at a nano- to micro-liter scale [149]. The use of such microfluidic perfusion mechanisms in organ-on-a-chip models provides improved homeostatic activity through mimicking the flow of blood which not only provides nutrients, but also removes catabolic metabolites and waste products [150,151,152]. Therefore, brain- and microvessel-on-a-chip [65,153] and human organoids [154] have recently emerged as powerful tools for modelling the pathophysiology of various NDDs such as AD, PD, and HD since they enable a wide range of research applications, spanning the evaluation of disease progression, novel drug development, screening and non-invasive real-time monitoring of drug action [155,156,157].

## 4. Current Challenges and Future Perspectives

One of the major challenges facing the HTS in the discovery of promising “hits” for treating the NDDs is the limited capacity of the use of models to perfectly recapitulate the pathophysiological milieu of NDDs. The CNS is one of our body’s most heavily cellularized tissues, and the extracellular matrix, which occupies 20% of the CNS space, is an extremely significant additional layer of complexity. Therefore, current endeavors should indeed be targeted at developing new models and novel screening techniques that better recapitulate the in vivo physiologies [158]. The brain is the most sophisticated organ in the human body, summarized by theoretical physicist Michio Kaku who said “the human brain has 100 billion neurons, each neuron connected to 10,000 other neurons. Sitting on your shoulders is the most complicated object in the known universe” [159]. It is this complexity that means creating a model completely mimicking the physiological/pathophysiological conditions in the brain is not an easy nor straight forward task. However, recent breakthroughs in harnessing the advantages of the iPSCs in combination with the advancement in microfluidic systems and 3D culture models could lead to constructing brain-on-chip models that have a better ability in recapitulating pathophysiological condition(s) most closely related to those in NDDs patients [160]. For instance, currently, most in vitro models of NDDs are comprised largely from neuronal cells, therefore, their accuracy, and also complexity, would be increased through adding glial cells such as astrocytes, microglia, pericytes, and oligodendrocytes to better mimic the molecular and structural complexity [66,161,162,163]. Even after drug administration, the BBB can prevent the passage of over 98% of small molecule drugs and other therapies to the brain [66,164]. In addition, the BBB impairment is known to diminish its ability to prevent the peripheral immune cells from infiltrating the brain, so it is implicated in exacerbating the condition of patients with NDDs [66] such as AD and PD [165,166,167,168,169,170,171,172]. Therefore, future 3D models for NDDs should incorporate the BBB to more precisely model pathophysiological conditions and promote the targeted delivery of therapies, whilst lowering the potential for serious side-effects [151,173].

HTS is indispensable in the field of drug discovery for NDDs. However, the capability of HTS is not only restricted by the availability of human-relevant NDD models, but also limited by the quality and size of the library of the compounds screened in the HTS. Therefore, expanding the number of small molecules available for HTS increases the odds of discovering efficient disease-modifying drugs for the NDDs. Dynamic combinatorial chemistry (DCC) advancement, the introduction of cheminformatics to the pharmaceutical industry, along with the widespread use of artificial intelligence (AI) in the arena of drug discovery are expected to generate a huge number of compounds that may lead to more targeted drugs with activity in preliminary HTS assays [174,175,176].

Finally, in addition to the technical challenges and obstacles discussed previously, the elevated cost associated with the process of discovering a novel drug is one of the major factors that push the pharmaceutical industry away from the race to find novel drugs for NDDs. Interestingly, the recent advancements in exploiting AI and machine learning in pharmaceutics has shown enormous potential in making the process of novel drugs discovery cheaper and more effective [175]. Recently, a number of pharmaceutical companies have started to harness the power of AI through the development of algorithms in the quest for drug-structure prototypes within published research papers and curated databases. For example, Benevolent Bio Company (New York, NY, USA) is presently exploiting AI for discovering new ways to treat ALS. Researchers at Benevolent Bio identified 100 potential compounds for treating ALS, but the AI intervention meant that only five of these potential compounds were tested in patient derived neuronal cells. Researchers discovered that one of the five tested compounds had prominent activity in slowing the neurological symptoms of ALS in a mice model [177]. Therefore, with the aid of AI, the process of HTS assays for discovering a novel disease-modifying drug that targets NDDs is expected to be a less expensive and faster process in the very near future.

HTS informatics systems continue to embrace these new innovations and increasingly help extract HTS data more efficiently from the initial assays and subsequent platform analyses. In fact, researchers have also developed platforms to mimic multiorgan interactions that are not present in conventional tissue culture systems, using microfluidics with a co-culture system with the aim of studying the pharmacokinetics of drugs [178] or cell-based drug metabolism [179]. Although not yet applied to the CNS, “quasi-all-body” model systems may reveal novel key information in this field. Both fully automated robotic systems and dedicated workstations can be used in the automation of HTS units. There is an ongoing conversation around the most reliable and cost-effective automated HTS devices [180]. The selection between full robotic systems and assay specific workstations depends on multiple factors such as budget, shift patterns, and the available workforce.

In conclusion, NDDs are set to become a modern “silent epidemic” placing a major healthcare burden on countries with aging populations. Emerging advances in HTS combined with major developments in disease modelling and computational tools have become fundamental in tackling this unmet clinical demand and will help towards achieving more personalized treatments and effective precision medicine in the foreseeable future.

## Data Availability

Not applicable.
